# Efficacy of copper-impregnated antimicrobial surfaces against *Clostridioides difficile* spores

**DOI:** 10.1017/ice.2024.219

**Published:** 2025-02

**Authors:** Thanuri Navarathna, Piyali Chatterjee, Hosoon Choi, John D Coppin, Brandon Corona, Emma Brackens, Lynn Mayo, Munok Hwang, Marjory Williams, Morgan Bennett, Chetan Jinadatha

**Affiliations:** 1 Department of Research, Central Texas Veterans Health Care System, Temple, TX, USA; 2 Department of Medicine, Central Texas Veterans Health Care System, Temple, TX, USA; 3 Department of Medical Education, College of Medicine, Texas A&M University, Bryan, TX, USA

## Abstract

**Objective:**

*Clostridioides difficile* (*C. difficile*) is one of the most common causes of healthcare-associated infections (HAIs). Elimination of *C. difficile* spores is difficult as they are resistant to common hospital-grade disinfectants. Copper-impregnated surfaces provide continuous reduction of multiple pathogens, potentially lowering the risk of infections. This manuscript aims to evaluate the efficacy of copper-impregnated surfaces on *C. difficile* spores.

**Methods:**

Control (no copper) coupons and copper coupons containing 20% copper-oxide were inoculated with *C. difficile* spore loads ranging from 10^5^ to 10^7^ spores, with or without 5% fetal bovine serum soil load. After 4 hours of contact time, the *C. difficile* spores were recovered, plated on *C. difficile* growth media, and colony forming units were counted. The efficacy of copper (log_10_ kill) was estimated using a Bayesian latent variables model.

**Results:**

After 4 hours, unsoiled copper bedrail and copper table coupons at mean spore inoculation resulted in a 97.3% and 96.8% reduction in spore count (1.57 and 1.50 log_10_ kill, respectively). That of soiled bedrail and table coupons showed a 91.8% and 91.7% reduction (1.10 and 1.10 log_10_ kill, respectively).

**Conclusions:**

Copper coupons can substantially reduce *C. difficile* spores after 4 hours, but results vary depending on the initial spore concentration and presence or absence of organic material. Higher initial spore loads or excess organic material may prevent spores from contact with copper surfaces, thus decreasing kill efficacy. Continuous sporicidal effect of copper-impregnated surfaces may decrease spore burden and help prevent transmission of spores.

## Introduction

Healthcare-associated infections (HAIs) cause great burden on the healthcare system through increased length of stay, cost, and mortality.^1^ Contaminated healthcare surfaces play a major role in the spread of drug-resistant pathogens from surfaces to patients via healthcare worker hands. Further, these pathogens can survive on these high-touch surfaces for several months.^2,3^ *Clostridioides difficile* (*C. difficile)* is a spore-forming bacterium that is one of the leading causes of HAIs in the United States, with an incidence rate of 54 cases per 100,000 people as of 2022.^4,5^ *C. difficile* spores exist in the environment for several months, making it an important pathogen in surface-related transmission of HAIs.^2^ While prior studies have demonstrated that improved surface disinfection can decrease the environmental microbial burden and probability of HAIs,^2,6^ elimination of *C. difficile* spores is difficult as they are resistant to hospital-grade disinfectants such as quaternary ammonium-based chemicals.^7^ Sporicidal chemical disinfectants have been shown to effectively eliminate *C. difficile* spores on surfaces after appropriate contact time.^7,8^ Although disinfectants such as sodium hypochlorite, hydrogen peroxide with peracetic acid, and hypochlorous acid are known sporicidal agents, residual contamination may persist due to insufficient dwell times, cross-contamination, and human error.^9,10^ Previous studies using no-touch disinfection (NTD) technologies have shown a >6 log reduction in *C. difficile* spores using hydrogen peroxide vapor, which has also been associated with lower incidences of *C. difficile* infections in a hospital environment.^11,12^ Ultraviolet sanitation has been found to decrease spore count by 4.4 log_10_ colony forming units (CFU) after 5 seconds of exposure.^13,14^ Current decontamination approaches apart from chemical disinfection that include NTD technologies such as ultraviolet sanitation/disinfection^15^ or hydrogen peroxide vapor cannot be used while a patient is occupying a room. Chemical disinfection and NTD technologies, therefore, represent episodic cleaning events usually either performed routinely and/or after patient discharge, allowing for rapid recontamination in between discharges.^16,17^

Copper alloy-based and copper-impregnated solid surfaces have emerged as promising methods of continuous surface decontamination in recent literature.^18–22^ In addition, the antimicrobial activity of copper-impregnated hospital linen to reduce *C. difficile* infections has been evaluated.^23^ The action of copper as a biocidal agent is mainly mediated through different redox states of copper such as Cu(II) and Cu(III) that can cause cellular damage leading to a loss of membrane potential and cytoplasmic content, and production of reactive oxygen species.^24^ While it has been shown that copper alloys can provide continuous reduction of *C. difficile* spores^25^ and infections due to *C. difficile*,^26^ the efficacy of copper-impregnated antimicrobial surfaces on *C. difficile* spores has not been previously evaluated. This study examines the sporicidal effect of copper-impregnated surfaces on *C. difficile* spores in a laboratory setting.

## Methods

The study was conducted at the Central Texas Veterans Health Care System following IRB approval.

### Preparation of C. difficile spores

Nontoxigenic *C. difficile* strain VPI 11186 was purchased (ATCC® strain 700057™, with complete whole genome sequencing data available) and streaked onto anaerobic blood agar plates and grown at 35°C in anaerobic conditions for 10 to 14 days for sporulation. *C. difficile* spore purification was completed using Environmental Protection Agency protocol (EPA protocol # EPA MLB SOP MB-28) and resulting purified spores were stored at −80°C.

### Experiments with copper-containing coupons

**Figure 1.**
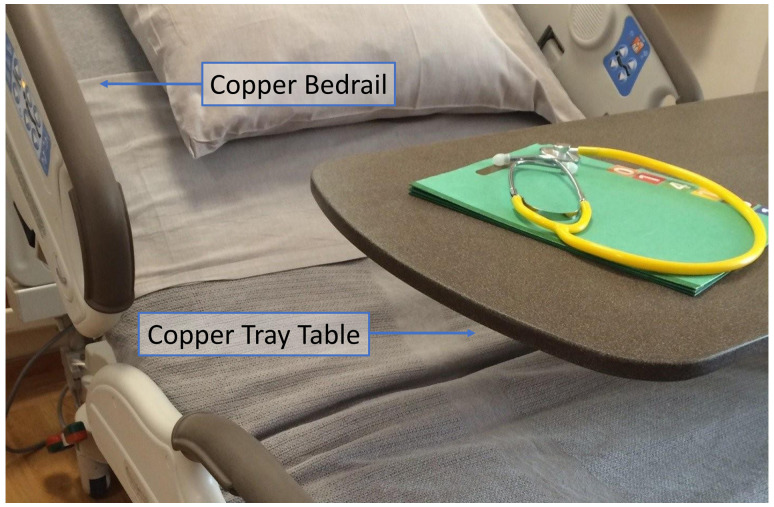
Copper-impregnated surfaces molded to cover bedside arm rails and tray tables.

Each experiment consisted of taking a *C. diff*icile spore stock sample and making a 1:10, 1:100, and 1:1000 dilution. Samples were taken from each of these three dilutions and pipetted and spread onto the surface of three, circular, 2 cm^2^ coupons, for each of the four materials (stainless steel, non-copper control material, 20% copper-impregnated bedrail material, and 20% copper-impregnated table material). Control and copper-impregnated coupons were received from the company (EOS^Cu^ Surfaces, Norfolk, VA) that manufactures the actual copper-impregnated surfaces as shown in Figure 1.

Thus, 36 coupons were used per experiment (3 coupons *4 materials *3 dilutions = 36 coupons per experiment). Four experiments were conducted in total, two experiments with and two without a 5% fetal bovine serum soiling solution in the buffer, to approximate organic material in clinical settings. After 4 hours of contact time, the *C. difficile* spores were retrieved from the coupons via vortexing in phosphate-buffered saline with Tween-80 (PBS-T) for 1 minute. The *C. difficile* spores were plated on CHROMID® *C. difficile* media plates at varying dilutions ranging from 10^0^ to 10^5^ and grown at 35°C in anaerobic conditions for 48 hours. The observed CFUs on the media plates were then counted. More than 330 CFU was recorded as too numerous to count. The exact concentration of initial spore stock solution was measured with serial dilution and counting CFUs as described above. The methodology for measuring the antimicrobial activity of the copper-impregnated coupons was based on an EPA protocol for the evaluation of bactericidal activity of hard, non-porous copper-containing surface products,^27^ with modifications made as described above.

Additional experiments were done with Ethylenediaminetetraacetic acid (EDTA), which can neutralize copper ions, to confirm the mechanism of biocidal effect of copper on *C. difficile* spores was through direct contact rather than the leeching of copper ions in the PBS-T. Copper ions were measured using a Copper Detection Assay Kit (Abcam catalog# ab252901).

### Statistical analysis methods

The protocol was written into a full joint probabilistic model for the process as a Bayesian latent variables model. The error propagated through serial dilutions and realized as variation in observed spore counts on media plates was modeled using a Poisson distribution. Samples from the stock solution and samples for coupon inoculation from the 1:10, 1:100, and 1:1000 dilutions were modeled as deviations arising from a Gaussian distribution with latent mean and standard deviation. Information that plates deemed ‘too numerous to count’ by the laboratory technicians had counts greater than 330 colonies (i.e. censoring) was included in the model likelihood (https://mc-stan.org/docs/stan-users-guide/truncation-censoring.html#integrating-out-censored-values).

The plate counts from the serial dilution straight plating were included in the model to help inform the latent spore stock amount. Thus, the full data from all media plates was used in the model, increasing the precision of all estimated parameters. A linear function with parameters for the surface material, soiled solution, and initial spore inoculation, modeled the spore loss on the four coupon materials. The estimated difference in spore loss between the control and copper coupons gave the estimated treatment effect of the copper coupon. The models were fit in Stan using rstan version 2.21.2 in R version 3.6.3.^28^ Results are presented as means and 95% central quantiles of the Markov Chain Monte Carlo samples from the posterior probability distribution for the parameters.

## Results

**Figure 2.**
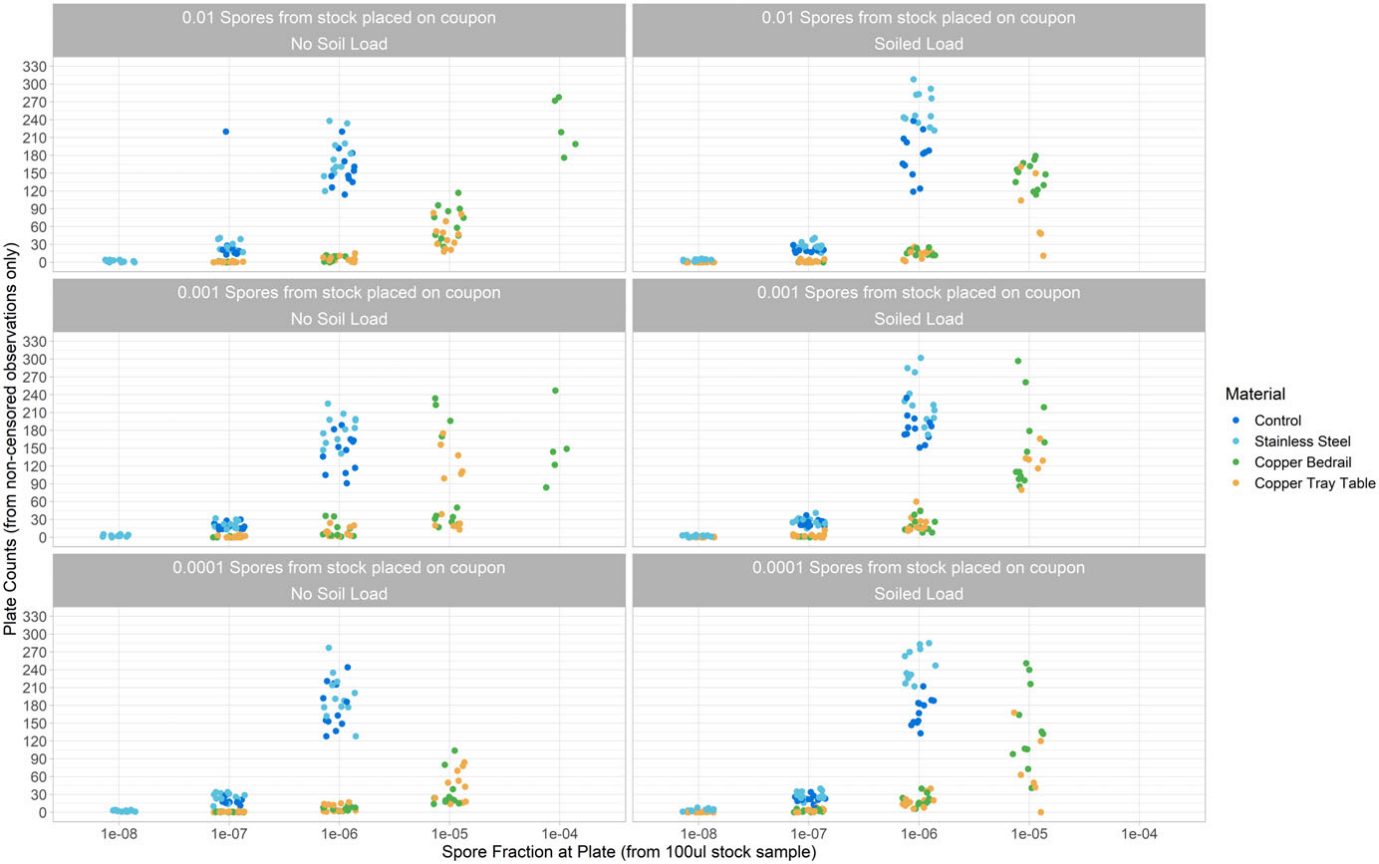
Non-censored plate counts for each dilution.

The estimated number of spores in the four samples from stock solution used in these experiments was 8.57 (8.54–8.59), 8.36 (8.33–8.40), 8.41 (8.38–8.45), and 8.43 (8.40–8.46) log_10_. The estimated error in the process of sampling from diluted stock and spreading the spore solution onto the coupon was 0.24 (0.21–0.27) log_10_ standard deviations. The non-censored plate counts for each dilution are shown in Figure 2.

**Figure 3.**
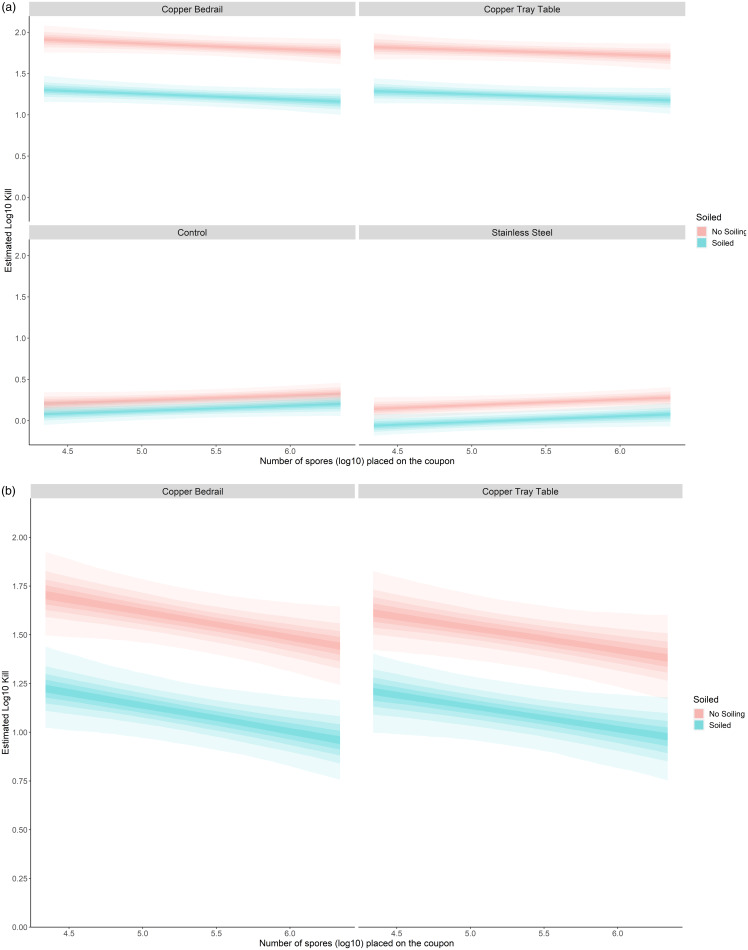
(a) Spore loss on each material after 4 hours with and without soiling. (b) Spore loss on each copper material after 4 hours with and without soiling.

The spore loss (Figure 3a) on each material after 4 hours at the mean spore inoculation in an unsoiled solution was 1.84 (1.72–1.97), 1.77 (1.65–1.89), 0.21 (0.10–0.32), and 0.27 (0.16–0.37) log_10_ loss for the copper bedrail, copper table, stainless steel, and control material, respectively. The treatment effect of the copper can be calculated by subtracting the spore loss of the control material from the spore loss for the copper materials due to desiccation. Thus, the estimated treatment effect of the copper in the copper bedrail material at the mean spore inoculation for an unsoiled solution was 1.57 (1.42–1.73) log_10_ kill, and for the copper table under the same conditions was 1.50 (1.34–1.67) log_10_ kill (Figure 3b). For the soiled solution, the estimated treatment effect was 1.10 (0.93–1.25) log_10_ kill on the bedrail and 1.10 (0.92–1.25) on the table. This represents a 97.3% (96.2%–8.1%) and 96.8% (95.4%–97.8%) reduction in the number of spores placed on the coupon after 4 hours due to the copper in the bedrail and table material for unsoiled solution, respectively. For soiled solution, there was a 91.8% (88.3%–94.3%) and 91.7% (88.1%–94.3%) reduction in the number of spores for the bedrail and table material, respectively.

**Figure 4.**
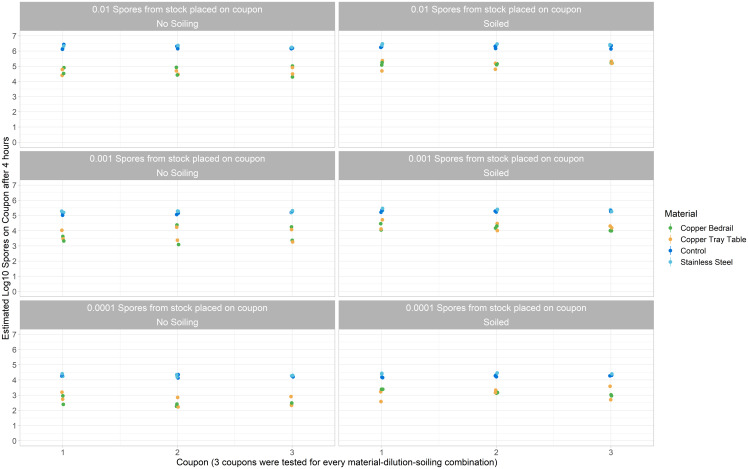
Estimated log_10_ number of spores on each coupon after 4 hours.

The spore loss varied depending upon the inoculation amount on the coupon and whether the solution included soiling (see Figure 3a). For the stainless steel and control material, the log_10_ spore loss increased slightly with increasing inoculation load on the coupon, although this effect was not within the 95% uncertainty interval. For each standard deviation increase in the inoculation load, there was a 0.07 (0.15 to −0.02) and 0.06 (−0.14 to −0.03) log_10_ increase in the spore loss for the stainless steel and control material, respectively. This process was reversed for the copper materials, where the log_10_ spore loss decreased with increasing inoculation load on the coupons, with a 0.07 (−0.02 to 0.17) and 0.06 (−0.04 to 0.16) log_10_ decrease in the spore loss effect for the copper bedrail and table, respectively. Thus, in terms of the estimated treatment effect of copper in the copper material, the overall log_10_ kill effect decreased with increasing spore inoculation load on the coupon by 0.13 (−0.00 to 0.27) and 0.11 (−0.01 to 0.25) for each standard deviation increase in the spore inoculation load on the coupon for the copper bedrail and table, respectively (see Figure 3b). The soiled solution also decreased the estimated log_10_ kill effect of the copper in the copper material, with log_10_ kill for the soiled solution decreased by 0.48 (0.26–0.71) and 0.41 (0.19–0.63) log_10_ for the copper bedrail and table, respectively (see Figure 3b). The estimated log_10_ number of spores on each coupon after 4 hours is shown in Figure 4.

The supplemental EDTA experiments showed that *C. difficile* spores are killed by copper at about the same efficacy regardless of the presence of EDTA.

## Discussion

Our results demonstrate that copper-impregnated coupons substantially reduced *C. difficile* spores after 4 hours, but the magnitude of reduction varied depending on the initial spore concentration and presence or absence of organic material. Higher initial spore loads, or excess organic material prevented the spores from coming in direct contact with the copper-impregnated surface thus decreasing the kill efficacy. After 4 hours, this study showed about a 97% and 92% reduction in spore count for unsoiled and soiled copper bedrail and copper table coupons at mean spore inoculation, respectively. Similarly, copper alloy-based solid surfaces have known antimicrobial activity with notable bacterial colonization reduction in both the laboratory and clinical settings.^18^ In comparison to copper-impregnated surfaces, one study showed copper alloys with concentrations of copper varying between 65% to 100% content resulted in roughly a 2-log reduction in *C. difficile* spores after three hours, when compared to stainless steel alloy.^25^ Another similar study showed copper alloys containing at least 97% copper resulted in a significant (∼2.67 to 2.96) log reduction in three hours against germinating *C. difficile* spores, while stainless steel alloy did not show any such antimicrobial activity.^29^ Previously the copper alloy surfaces (percentage of copper >70%) were shown to be effective in reduction of survival of both vegetative and spores of *C. difficile.* ^25^ Copper-impregnated surfaces (percentage of copper 20%) used in our study was able to achieve high reduction percentage in *C. difficile* spore counts at 4 hours. As shown by the supplemental EDTA experiments, *C. difficile* spores were killed by copper at about the same efficacy regardless of the presence of EDTA, suggesting that the method by which copper kill spores is primarily through direct contact with copper impregnated in the coupon rather than leached copper ions.^30,31^

Copper-impregnated surfaces used in this study are prepared with uniform copper oxide content (Figure 1) are not a coating or a spray. This is an advantage as even with surface wear the same amount of active copper is still presented to the environment, resulting in easy maintenance. The mixtures of copper oxide and substances such as polymers can be heated and poured into a mold and conformed into any desired shape; a property copper alloys do not have as the machinability of copper confines its use to relatively simple geometric forms.^32^ If copper-impregnated surfaces break, they would need to be remolded and installed; however, if molded and installed correctly the surfaces are not prone to breakage. Copper-impregnated surfaces can be cleaned with all commonly used hospital disinfectants without change to the antimicrobial or physical properties. Recent study suggests that the use of some chemical disinfectants used for routine disinfection (bleach, activated hydrogen peroxide) over prolonged time periods can affect the surface characteristics and level of antimicrobial activity of copper-impregnated surfaces.^33^ In contrast, copper alloys may produce patina and discoloration after repeated contact, although this does not affect its antimicrobial properties.^34^

A single-site quasi-experimental study assessing the effect of using copper-impregnated surfaces on HAIs incidence in a Virginia hospital showed a 78% decline in HAIs due to MDROs or *C. difficile* and 83% fewer cases of *C. difficile* infection after the introduction of copper-impregnated surfaces and copper-infused linens.^35^ The study design may have contributed to higher percentages of HAI decline and may not be solely attributed to the introduction of copper alone. Overall evidence suggests that the sporicidal effect of copper-impregnated surfaces may have the potential to reduce transmission of spores in a continuous manner and prevent HAIs in a clinical setting, supplementing episodic and terminal cleaning methods of hydrogen peroxide vapor, ultraviolet sanitation, and other sporicidal disinfectants.

Previous studies *on C. difficile* spore contamination recovered from the environmental surfaces in a hospital environment are known to vary between <1 to 2 log_10_.^36–38^ Few studies reported higher spore counts from >200 colonies,^39^ and up to 1,300 colonies.^40^ Our laboratory-based studies were performed based on EPA disinfectant standards, but the spore load found in clinical scenarios as mentioned above is expected to be much lower than the concentration with which we tested the copper-impregnated coupons. We therefore anticipate the efficacy of the *C. difficile* log kill will be higher in the copper-impregnated surfaces.

This study was designed to examine the efficacy of copper-impregnated surfaces on *C. difficile* spores with and without the presence of organic material. However, there are some limitations. We did not assess the proportions of copper in the solid surface that affects kill rate positively or negatively nor did we assess its effects on contact time. Hence these results cannot be compared to effect sizes of materials with different composition inclusive of higher or lower proportions of copper and organic material. Other limitations include the limited number of copper-impregnated surfaces that were evaluated, and the impact of repeated use of disinfectants was also not evaluated.

These findings demonstrate that the copper-impregnated surfaces we tested reduce *C. difficile* spore count by 92% to 97% thus providing additional disinfection opportunity in between episodes of daily disinfection and terminal room disinfection in acute care settings by delivering continuous sporicidal activity to lower *C. difficile* bioburden.

## Supporting information

Navarathna et al. supplementary material Navarathna et al. supplementary material

## Supplementary material

To view supplementary material for this article, please visit https://doi.org/10.1017/ice.2024.219
